# Tumor-associated antigen CAPERα and microvessel density in hepatocellular carcinoma

**DOI:** 10.18632/oncotarget.7707

**Published:** 2016-02-25

**Authors:** Liping Dai, Xuan-Xian Peng, Eng M. Tan, Jian-Ying Zhang

**Affiliations:** ^1^ Department of Biological Sciences & NIH-Sponsored Border Biomedical Research Center, The University of Texas at El Paso, El Paso, TX 79968, USA; ^2^ School of Life Sciences, Sun Yat-sen University, Guangzhou 510006, China; ^3^ Department of Molecular and Experimental Medicine, The Scripps Research Institute, La Jolla, CA 92037, USA

**Keywords:** CAPERα, tumor-associated antigen, hepatocellular carcinoma, cancer biomarkers, tumor angiogenesis

## Abstract

**Purpose:**

CAPERα, a tumor-associated antigen, was identified from a cDNA clone with autoantibody from a patient with hepatocellular carcinoma (HCC). It has been implicated, by way of alternative splicing of VEGF pre-mRNA, in the regulation of microvessel formation in Ewing's sarcoma. In this study, we looked for possible association of alterations in CAPERα with microvessel density in HCC.

**Methods:**

Enzyme-linked immunosorbent assay using recombinant CAPERα as antigen were used to detect antibody against CAPERα. Immunohistochemistry (IHC) on liver sections was performed to analyze expression profiles of CAPERα, VEGF and CD34 in HCC and control tissues and was further used to assess the correlation of expression among CAPERα, VEGF and CD34 in HCC development.

**Results:**

Autoantibody to CAPERα was highest in HCC (22/76, 28.9%), not detected in prostate cancer (0/79) and at 3.4% (3/88) in breast cancer. In immunohistochemical analysis of grades II and III HCC tissues, significantly decreased immunostaining for CAPERα was observed and this correlated directly with decreased immunostaining for VEGF (*R*=0.534, *P*=0.0003). Using CD34 immunostaining for detecting newly formed microvessels, strong staining was observed in grades II and III HCC. Normal liver sections, all of which have high expression of CAPERα were totally negative for CD34 immunostaining. A significant inverse correlation was seen between CAPERα and CD34 immunostaining (*R*=−0.481, *P*=0.0012).

**Conclusions:**

Decreased expression of CAPERα appears to be correlated with appearance of microvessels. It would be of interest to elucidate the cause of altered CAPERα since new formation of microvessels is important in progression of HCC.

## INTRODUCTION

Hepatocellular carcinoma (HCC) is the third most common cause of cancer-related deaths worldwide [1] and is prevalent in countries with high rates of viral hepatitis. Unresolved viral hepatitis often leads to chronic hepatitis or liver cirrhosis and in these conditions, approximately one-third of patients will ultimately develop HCC over a period of several years [2]. This interval is a situation favorable for investigation of altered biological pathways or mechanisms which lead to tumorigenesis. We first observed that some patients with chronic hepatitis and liver cirrhosis developed humoral autoimmune responses to cytoplasmic and nuclear proteins, manifested as circulating autoantibodies reactive with tumor-associated antigen (TAAs) [3]. Further observations showed that in many patients there were autoantibodies present in the circulation which pre-dated the clinical detection of HCC and with conversion to cancer, autoantibodies of novel specificities appeared [3-5]. With the notion that the novel humoral immune responses might be antigen-driven, such as by structurally or functionally altered TAAs, HCC sera were used to screen cDNA expression libraries to identify cell components which might be participating in activated tumorigenesis pathways. A presumptive TAA which was isolated using serum autoantibody from a patient with HCC was a cellular protein of 64 kDa (530 amino acids) called HCC1.4 [6]. The deduced amino acid sequence contained an arginine/serine-rich (RS) domain and three ribonucleoprotein consensus sequence domains. Such motifs are found in RNA-binding proteins and in mRNA splicing factors.

While searching for transcriptional co-regulator proteins using the yeast two-hybrid screening assay and activating signal cointegrator (ASC-1) as bait, Jung et al isolated a protein which was a specific transcriptional coactivator of AP-1, ERα and ERβ which they called CAPERα [7]. CAPERα was identical to HCC1.4. Another study by Dowhan et al [8] showed that CAPERα/HCC1.4 regulated both steroid hormone receptor-mediated transcription and alternative splicing and further showed that CAPERα was involved in alternative splicing of VEGF mRNA. Thus CAPERα/HCC1.4 served the dual role of hormone receptor-regulated transcription and pre-mRNA splicing (in the interest of uniformity CAPERα will be used henceforth). Evidence that CAPERα was involved in tumorigenesis acting as a tumor suppressor was shown by Dutta et al [9]. Recently, Huang et al [10] showed that upregulation of CAPERα expression led to higher ratios of the 189/165 isoforms resulting in decreased tumor growth and decreased tumor vessel density in Ewing's sarcoma and downregulation of CAPERα expression had the opposite effect and induced increased tumor growth and tumor vessel density, related to higher ratios of 165/189 isoforms instead of 189/165 isoforms.

In view of these recent observations showing that altered function of CAPERα promoted tumorigenesis, we were interested in determining the frequency of anti-CAPERα autoantibody responses in HCC and other solid tumors and whether it might be possible to show the relationships existing between CAPERα and VEGF expression and whether they were associated with microvessel density in HCC.

## RESULTS

### Prevalence of autoantibodies against CAPERα

**Figure 1:**
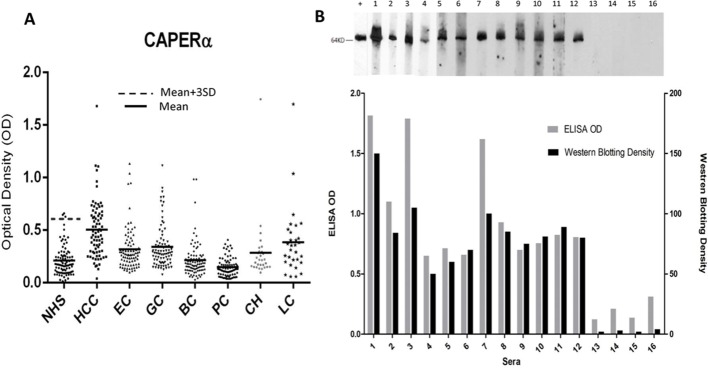
Titers of autoantibodies to CAPERα in human sera determined by ELISA and comparison of Western blotting and ELISA assays for anti-CAPERα **A.** The range of autoantibody titers to CAPERα expressed as absorbance units in enzyme immunoassay (ELISA). NHS: normal human sera, HCC: hepatocellular carcinoma, EC: esophageal cancer, GC: gastric cancer, BC: breast cancer, PC: prostate cancer, CH: chronic hepatitis, LC: liver cirrhosis. **B.** Upper figure: Murine monoclonal antibody was used as the positive control in Western blotting. Lane 1: chronic hepatitis serum with ELISA positive result, lanes 2-4: liver cirrhosis sera with ELISA positive results, lanes 5-12: representative HCC sera and lanes 13-16: NHS. Lower figure: Bar graphs showing ODs in ELISA paired with Western blotting intensity determined by densitometric scanning.

Purified recombinant CAPERα was used as the antigen in ELISA to determine prevalence of autoantibody in various cancer sera (Table 1). The cut-off OD value used was mean+3SD of 89 NHS sera. Anti-CAPERα was significantly higher in HCC (28.9%) and gastric cancer (10.7%) sera than in NHS (3.4%). Other tumors like breast (3.4%) and esophageal cancer (6.0%) did not show statistically significant differences from NHS. In prostate cancer, no serum out of 79 cases examined was positive. Of interest was liver cirrhosis, a condition with predisposition for development of HCC, where the prevalence was 10.0%. The raw OD values of the ELISA assay are shown in Figure 1A where it can be seen that in some conditions like gastric, esophageal, breast cancer and liver cirrhosis, there were several patients with high reactivity with CAPERα in spite of lack of significant statistical difference with normal sera at the group level. Figure 1B shows in bar graphs, the pairing of ELISA OD readings with densitometric scanning readings of Western blotting bands. The Western blotting assay corresponded well with ELISA and confirmed that the enzyme immunoassay method was not also registering reactions with other proteins which could have been in the recombinant antigen preparation.

**Table 1:** Frequency of antibodies against CAPERα in human sera by ELISA

Type of sera	N	Antibody to CAPERα (%)
Normal human sera	89	3(3.4)
Hepatocellular carcinoma	76	22(28.9)**
Esophageal cancer	84	5(6.0)
Gastric cancer	84	9(10.7)*
Breast cancer	88	3(3.4)
Prostate cancer	79	0(0.0)
Chronic hepatitis	29	1(3.5)
Liver cirrhosis	30	3(10.0)

Cutoff value: mean+3SD of NHS; *P*-value relative to NHS

* *P*<0.05

** *P*<0.01.

### Expression of CAPERα in different cancer cell lines

**Figure 2:**
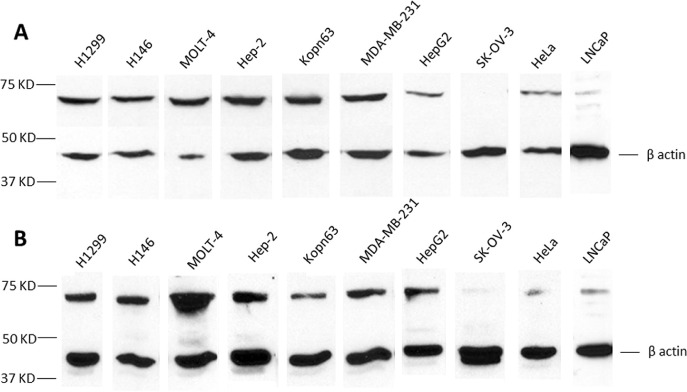
Analysis of tumor cell lines for CAPERα expression Monoclonal antibody **A.** and HCC serum NK **B.** were used in Western blotting to determine the relative expression of CAPERα in the following tumor cell line lysates: H1299, H146, MOLT-4, HEp-2, KOPN 63, MDA-MB-231, Hep G2, SK-OV-3, HeLa and LNCaP.

The observations above showed that the majority of autoimmune antibody responses to TAAs are not specific or restricted to any one species of tumor but might demonstrate higher prevalence in some tumors than others. We randomly selected 10 well-established human tumor cell lines derived from 9 different organs and made solubilized extracts from these cell lines to determine expression of CAPERα in these cells. Most tumor cell lines showed expression of CAPERα, some with robust expression but others with weak expression like LNCaP and HeLa or undetectable expression like in the ovarian cell line, SK-OV-3 (Figure 2). It could be of some interest if the low expression of CAPERα in the prostate cell line LNCaP was related to the absence of autoantibody to CAPERα in prostate cancer, but this could be a co-incidence with this particular cell line. Panel A of this figure was probed with the murine monoclonal antibody to CAPERα and panel B with human HCC serum which was previously used for identifying CAPERα and the β actin reaction was the internal control for each solubilized cell extract. Very similar reactivities such as detection of high or low abundance of the antigen in different cell lines were seen between murine monoclonal antibody and human HCC serum.

### Immunohistochemistry to evaluate expression and distribution of CAPERα in HCC and normal liver sections

Many TAAs have been identified with autoantibodies from cancer patients and although there is evidence that the stimulus for the autoimmune response is antigen driven [17-20], much remains to be elucidated concerning the cellular pathways which lead from an original TAA-initiated antigenic stimulus to overt carcinogenesis. An opportunity for some insight on this issue might come from the recent reports that CAPERα is a tumor suppressor protein and is capable of alternative mRNA splicing function on VEGF, and depending on ratios of splice products, this in turn regulates formation of microvessels in tumors such as Ewing's sarcoma [6-10]. The objective was to evaluate changes, if any, between HCC and normal liver with respect to expression of CAPERα using the murine monoclonal anti-CAPERα as the detecting reagent in immunohistochemistry.

**Figure 3:**
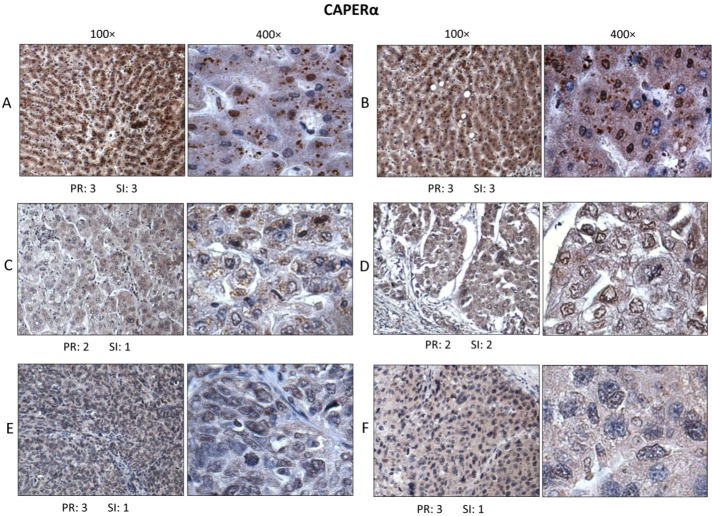
Immunohistochemistry to determine expression and localization of CAPERα Section **A.** was from a normal liver, section **B.** from a non-cancer area in liver that was adjacent to tumor, sections **C.** and **D.** were from two different grade II HCC and sections **E.** and **F.** from two different grade III HCC. At higher magnification (400×), immunostaining was seen to involve the majority of hepatic cell nuclei and cytoplasm. The positive rate (PR) of immunostaining and staining intensity (SI), were evaluated based on the Yu method [16].

In Figure 3, in normal liver (Figure 3A), hepatic cells reacted strongly, with both cytoplasmic and nuclear staining (dark brown color of diaminobenzidine). In the cytoplasm, CAPERα immunostaining was both diffuse and in the form of small particles (seen best at 400×). In the tumor-adjacent histologically normal liver (Figure 3B), immunostaining was very similar. It was noted that not all hepatic cell nuclei were positively stained. The positive rate (PR) and staining intensity (SI) of cells reactive with antibody were given numerical ratings according to methods used by several other investigators [16, 21, 22]. In grade II HCC (Figure 3C and 3D), both positive rate and staining intensity for CAPERα staining were reduced in tumor cells compared to staining of normal hepatic cells in Figure 3A and 3B. A feature which was consistently observed was not only reduction in positive rate and intensity of staining for CAPERα but also absence of particulate cytoplasmic immunostaining in almost all grade II and III HCC cells. This was replaced by a diffuse cytoplasmic staining. In grade III HCC (Figure 3E and 3F), there was further reduction in intensity of CAPERα immunostaining in both nuclei and cytoplasm of cancer cells.

### Immunohistochemistry to evaluate expression and distribution of VEGF

**Figure 4:**
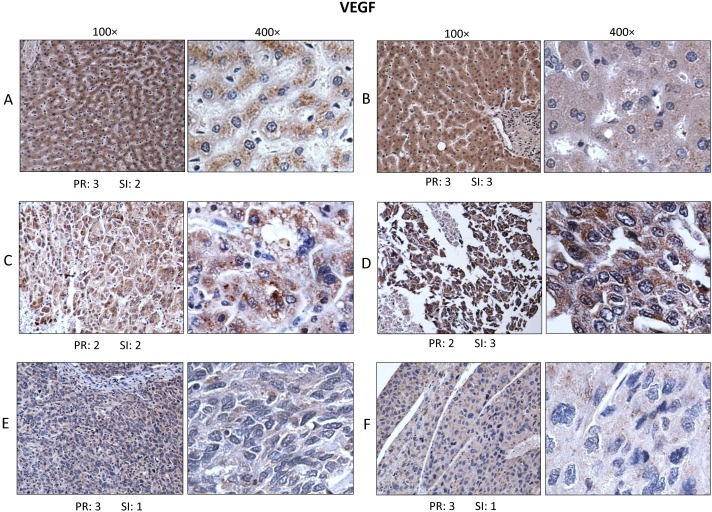
Immunohistochemistry for VEGF Sections from the same normal and HCC tissues as in Figure 3 were used for immunostaining using rabbit polyclonal antibody to VEGF and performed under identical conditions. Immunostaining for VEGF was restricted to cytoplasm of hepatic cells without any detectable nuclear staining. Cytoplasmic expression of VEGF in normal liver could be granular **A.** or diffusely dispersed **B.** In grade II HCC, VEGF immunostaining was variable in intensity and continued to be localized in the cytoplasm **C.** and **D.** In grade III HCC tissues, immunostaining was generally more reduced in intensity as in **E.** and **F.**

A second set of HCC and normal liver sections from the same subjects as in Figure 3 were used in this study and the detecting reagent was a polyclonal antibody raised in rabbits against an N-terminal peptide of VEGF-A of human origin (see Methods). The antibody was reactive with 189, 165 and 121 amino acid splice variants of VEGF. Immunostaining for VEGF was restricted to the cytoplasm of normal hepatocytes and of grade II and III HCC cells with absence of nuclear staining (Figure 4). The appearance of cytoplasmic staining could be granular as in 4A or diffuse as in 4B in normal and tumor-adjacent normal liver respectively. In grade II HCC, Figure 4C and 4D, it was apparent that immunoreactive VEGF was also exclusively in the cytoplasm but the staining intensity of HCC cells was not reduced compared to normal hepatocytes. A significant difference in VEGF immunostaining was seen in grade III HCC (Figure 4E and 4F) where cytoplasmic staining was further diminished compared to grade II HCC and normal liver.

### Immunohistochemistry to evaluate expression and distribution of CD34

**Figure 5:**
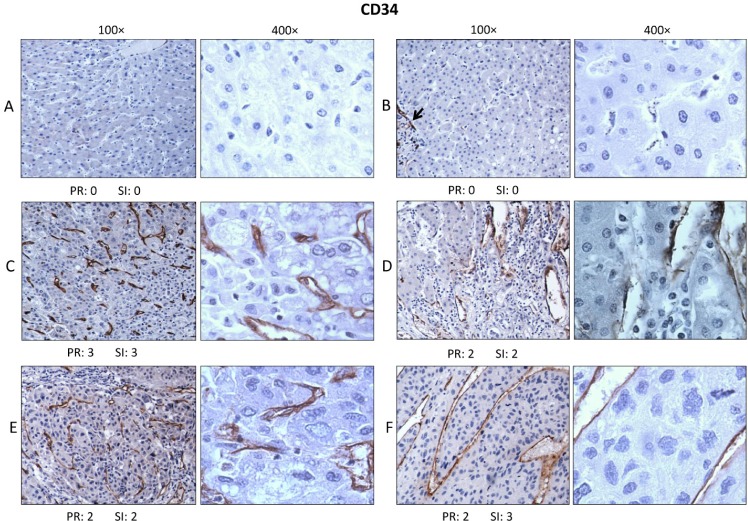
Immunohistochemistry for CD34 Murine monoclonal antibody to CD34 of human origin was used to determine expression of CD34 as marker of increased or newly formed microvessels in tissue sections from the same subjects as in Figures 3 and 4. In normal liver tissue **A.** and **B.** no CD34 immunostaining was detected, except for vessels in the portal triads (see arrow in B). However, in grades II **C.** and **D.** and III HCC **E.** and **F.** CD34 positive microvessels were expressed in many subjects, some associated with microvessels as in C and E and some showing ‘sinusoidal’-type staining as in F.

HCC has been widely recognized to be a highly vascular tumor, and the degree and density of vascularity has been associated with grade and aggressiveness of the tumor as well as the presence and extent of metastasis [23-28]. Several investigators have introduced methods to express in a semi-quantitative manner the degree or extent of microvessel formation (venules and capillaries) in HCC and other tumors using biomarkers such as CD34, CD105 and von Willebrand factor to assign numerical values to microvessel expression [29-33]. Since it has been shown that CAPERα is involved in alternative splicing of VEGF mRNA and changes in ratios of alternatively spliced VEGF isoforms lead to increased or decreased microvessel formation [8, 10], we examined microvessel density in HCC and normal liver using CD34 expression in microvessels as the surrogate marker. The tissue sections of HCC and normal liver were from identical subjects as in Figures 3 and 4). In normal liver (Figure 5A) and in histologically normal liver which was 1.5 cm distant from tumor (Figure 5B), there was no expression of CD34-positive microvessels. The arrow in Figure 5B points to a portal triad where there was CD34 staining but this is observed in all normal livers and does not represent newly formed microvessels [16]. In Figure 5C, CD34-positive microvessels are abundant in the 100× and 400× magnifications of a grade II HCC and there is no immunostaining of nuclei or cytoplasm in tumor cells. In Figure 5D, another grade II HCC, large and small lumens of microvessels are positive. Figure 5E shows CD34-positive microvessels in a grade III HCC and 5F shows sinusoidal staining of microvessels in another grade III HCC, a feature which has been previously observed in certain liver tumors [34]. In the seven normal liver tissues and five liver sections adjacent to tumors, no CD34-positive microvessels were detected.

### Relative expression of CAPERα, VEGF and CD34 evaluated by immunostaining

**Figure 6:**
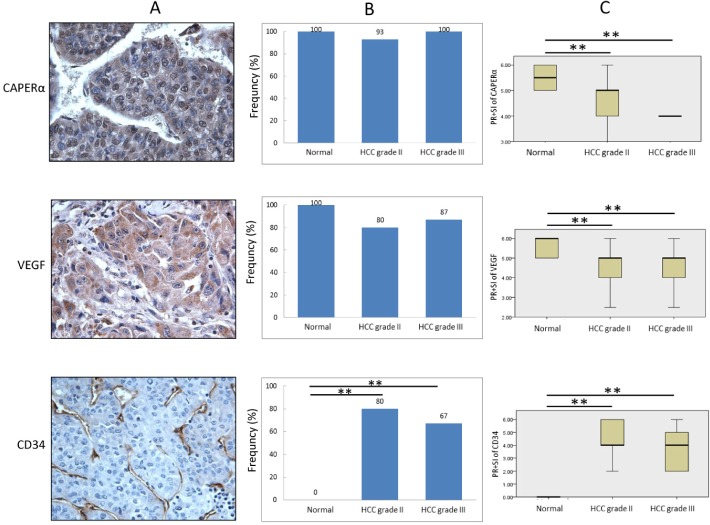
Comparison of expression of CAPERα, VEGF and CD34 in HCC and normal liver tissues The expression of three proteins in HCC was examined in 30 cases of HCC tissues (15 grade II and 15 grade III, respectively) and 12 normal or HCC adjacent normal tissues using IHC staining **A.** Frequency of high expression (PR+SI ≥ 4) of CAPERα, VEGF and CD34 in different conditions was compared by using χ^2^ test **B.** We also compared the expression level (PR+SI) of three proteins by Kruskal-Wallis test **C.** **: *P*<0.01 compared to normal control group.

Microscopic examination of tissue sections in immunohistochemistry gave the general impression that expression of CAPERα and VEGF was not an all or none phenomenon but rather one of different intensities. This was different from CD34 expression which was only seen in HCC but not in normal liver whether in normal or HCC-adjacent normal subjects. Figure 6A shows a fairly typical example of CAPERα immunostaining involving nucleus and cytoplasm of tumor cells, VEGF immunostaining involving only cytoplasm and CD34 immunostaining involving newly formed microvessels. Figure 6B, upper frame, shows the frequency of CAPERα expression in the three different groups of subjects, normal, grade II HCC and grade III HCC where no significant difference was detected between the groups. For VEGF immunostaining (Figure 6B middle frame), there was lower frequency in both grades II and III HCC compared to normals but not at statistically significant levels. In contrast, for CD34 immunostaining (Figure 6B lower frame), no histologically normal tissue was CD34-positive but 80 percent of HCC grade II and 67 percent of HCC grade III were positive. Figure 6C compares the extent and intensity of immunostaining for CD34 using combination of percent positivity and staining intensity (PR+SI) as described above. For CAPERα immunostaining (Figure 6C upper frame), HCC grades II and III both showed significantly reduced staining than normal and similarly for VEGF (Figure 6C, middle frame). For CD34 immunostaining (Figure 6C lower frame), because of the all or none nature of CD34 expression between normal and HCC, the figure shows negative expression of CD34 in normal and positive expression in grades II and III HCC. These data give support to the earlier observations that the relationship between CAPERα and HCC could be related to altered or reduced expression of CAPERα in HCC, followed by altered or reduced VEGF and expression of newly formed CD34-positive microvessels.

### Presence or absence of correlation in expression between CAPERα, VEGF and CD34

**Figure 7:**
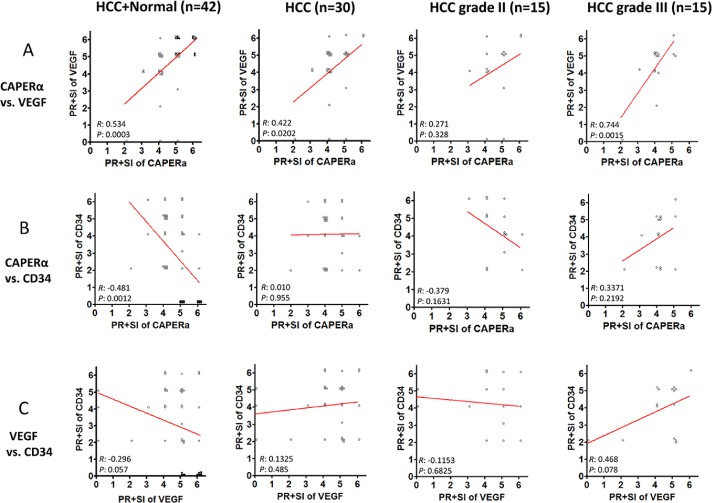
Co-expression of CAPERα, VEGF and CD34 in normal and HCC tissues Spearman's rank-order test was used to evaluate the co-expression of the biomarkers. The numerical assignment of the sum of PR and SI in immunohistochemistry was used in this analysis. The entire cohort, including 12 normal livers and 30 HCC livers were analyzed for possible correlations between CAPERα vs VEGF **A.** CAPERα vs CD34 **B.** and VEGF vs CD34 **C.** (filled circles represent normal liver and empty circles represent HCC livers).

It was of interest to determine whether there was any correlation in the increase or decrease of expression between pairs of the above cellular proteins. We used Spearman's Rank Correlation Coefficient and the sum of PR+SI for this analysis. Figure 7 panel A show that between CAPERα and VEGF, there was significant correlation in the entire cohort of HCC plus Normals (*R*=0.534, *P*=0.0003), in all HCC combined (*R*=0.422, *P*=0.0202) and in grade III HCC (*R*=0.744, *P*=0.0015). This was a positive correlation, higher expression of CAPERα was associated with higher expression of VEGF and vice versa. In grade II HCC, there was no correlation (*R*=0.271, *P*=0.328). A strong relationship was seen between CAPERα and CD34 in the entire cohort, where the *R* value was −0.481 and *P* value was 0.0012 (Panel B). In this instance, the correlation was inverse, the lower the CAPERα expression, the higher the CD34 expression and vice versa. No significant relationships were detected in the whole HCC cohort or between grades II and III (Panel B). A trend towards significance was seen between VEGF and CD34 in the entire HCC and normal cohort (*R*=−0.296, *P*=0.057, Panel C) but not in other groupings of subjects.

## DISCUSSION

CAPERα or HCC1.4 was isolated from a cDNA expression library using autoantibody from a patient with hepatitis B virus related HCC [6]. This patient had been under medical surveillance for many years because of liver cirrhosis and serial samples of serum were negative for anti-CAPERα until a few months prior to detection of malignancy [3]. Because of the timing of the autoimmune antibody response, we thought it was possible that was associated with tumorigenesis. At that time however, there were no clues as to how CAPERα might be involved in transformation from liver cirrhosis to malignancy, except for the fact that CAPERα contained sequence motifs possessed by RNA binding proteins and mRNA splicing factors, both of which have been involved in tumorigenesis pathways. Subsequent studies by many investigators have given insights into probable mechanisms. In looking for co-regulators of steroid hormone receptors, Jung et al [7] isolated a protein which was identical to HCC and called it CAPERα. Dowhan et al extended these studies and showed that CAPERα was capable of splicing VEGF into alternative spliced isoforms [8] and Dutta et al showed it had tumor suppressor activity by binding to the transactivating activation domain (TAD) of the oncogene v-Rel [9]. Recently, Huang et al gave further insights into the alternative splicing functions of CAPERα showing that in Ewing's sarcoma, variations in expression of CAPERα resulted in different ratios of spliced forms of VEGF, which in turn regulated expression of tumor microvasculature [10].

Our studies described here were guided to a large extent by the observations summarized above. Initially, we noted that the occurrence of autoantibodies to CAPERα was higher in HCC (28.9%) than in a number of other tumors and interestingly that in liver cirrhosis, a frequent precursor to HCC, the frequency was 10%, almost reaching a significant 0.05 P value. In immunohistochemistry, we were able to use a commercial murine monoclonal antibody to CAPERα for examining its expression in cancer and normal liver after showing that it reacted in a similar fashion to human autoantibody in the Western blotting of CAPERα differentially expressed by several tumor cell lines (Figure 2). When examining the expression of CAPERα using the murine monoclonal antibody, it was readily apparent that CAPERα was highly expressed in nuclei and cytoplasm of normal liver cells and the major difference with HCC cancer cells was a significant reduction in intensity of CAPERα immunostaining but no difference in expression frequency in cancer (Figure 6B, 6C). A similar reduction for immunostaining of VEGF was observed without significant reduction in frequency in HCC grades II and III. It would have been more informative if the anti-VEGF polyclonal antibody used was not polyreactive with both the 189 and 165 spliced isoforms of VEGF but was specific for one or the other isoform so that it could be ascertained whether the appearance of CD34-positive microvessels could be attributed to a higher ratio of VEGF 165/189 as described by Huang et al [10]. HCC is widely recognized to be a highly angiogenic tumor and is especially seen in poorly differentiated tumors [35-37] and VEGF is one of the factors stimulating angiogenesis [38, 39]. Our observations support the association between decreased CAPERα and appearance of CD34-positive microvessels but the association between decreased VEGF and CD34 is not apparent. This could be related to the lack of an immunological probe which could differentiate between the 189 and 165 isoforms of VEGF.

In studies of autoimmunity in cancer and cancer immunodiagnostics, an objective is to be able to track an event like finding of autoantibody to a tumor-associated antigen and continue with subsequent studies to determine how the tumor-associated antigen divulged by the autoantibody response is involved in tumorigenesis [40]. In the case of CAPERα, this has been possible through the convergence of studies by investigators in different fields. The studies reported here are preliminary and are based on immunological approaches. They need to be expanded with other approaches, combining both clinical and basic research, in order to have a more complete understanding of the pathway or pathways leading to HCC carcinogenesis.

## MATERIALS AND METHODS

### Serum samples

Sera from 76 patients with HCC, 29 patients with chronic hepatitis (CH), 30 patients with liver cirrhosis (LC), 84 patients with gastric cancer (GC), 84 patients with esophageal cancer (EC), 88 patients with breast cancer (BC), and 79 patients with prostate cancer (PC) were obtained from the serum bank of the Cancer Autoimmunity Research Laboratory at University of Texas (El Paso, Texas, USA). These sera originated from Xiamen University in China have been used in previous studies for autoantibodies to TAAs [11-14], but in a different context and with different panels of antigens. Eighty-nine normal human sera (NHS) were originally obtained from the serum bank in Autoimmune Disease Center at The Scripps Research Institute (La Jolla, CA, USA). All cancer patient sera were collected at the time of initial diagnosis, before they received any chemo- or radiation-therapy. Normal human sera were collected from working adults who had no obvious evidence of malignancy. The procedures related to human subjects were approved by the ethics committee of the Institutional Review Board of the University of Texas, El Paso. All patients who participated in the study provided written informed consent and were identified by number in the research, not by name, to protect the patients' private information.

### Expression and purification of CAPERα recombinant protein

Plasmid pET-CAPERα (pET-HCC1.4) carrying CAPERα cDNA was derived from the previous study [6]. Recombinant CAPERα was expressed in *E.coli* BL21 (DE3) cells and purified using nickel column chromatography. The protocol used for high-level expression and purification of 6× His-tagged proteins were performed as described (QIAGEN Inc., Valencia, CA, USA).

### Cell lines and cell extracts

Ten different tumor cell lines, including non-small cell lung carcinoma (H1299), small cell lung carcinoma (H146), T cell leukemia (MOLT-4), B cell leukemia (KOPN-63), laryngeal epidermoid carcinoma (HEp-2), breast cancer (MDA-MB-231), hepatocellular carcinoma (Hep G2), ovarian carcinoma (SK-OV-3), cervical carcinoma (HeLa), and prostate adenocarcinoma (LNCaP) were obtained from ATCC and cultured following the specific protocol for each cell line. Cells grown in monolayers were solubilized directly in Laemmli's sample buffer containing protease inhibitors. Solubilized cell lysates were analyzed using SDS-PAGE after brief sonication.

### Enzyme-linked immunosorbent assay (ELISA) to detect autoantibody to CAPERα

Purified recombinant CAPERα protein was diluted in phosphate-buffered saline (PBS) to a final concentration of 0.5ug/mL as the antigen for ELISA. Human serum samples diluted 1:200 were incubated as the first antibody. ELISA was performed as described previously [11, 15]. All positive sera were further confirmed by Western blotting.

### Western blotting

Purified recombinant CAPERα was run in SDS-PAGE and transferred onto nitrocellulose membrane. After pre-blocking in PBST with 3% non-fat milk for 1 h at room temperature, nitrocellulose membranes were cut in strips which were then incubated with patient sera diluted 1:200. HRP-conjugated goat anti-human IgG was applied as secondary antibody at a 1:4,000 dilution. Immunoreactive bands were detected using the ECL kit (Amersham, Arlington Heights, IL) according to the manufacturer's instructions.

### Immunohistochemistry (IHC) with tissue array slides containing sections of HCC and normal liver

Superfrost plus tissue slides which contained 30 paraffin-embedded HCC specimens (15 grade II and 15 grade III) and 12 normal liver tissue specimens (7 from normal livers and 5 from HCC-adjacent normal livers areas 1.5 cm away from the tumors) were purchased (Cat: LV804 and T032a; US Biomax, Inc., Rockville, MD, USA). These liver tissues were obtained at autopsy. They were used to determine whether there were any alterations in distribution of CAPERα, VEGF and CD34 in HCC compared with normal liver. The antiserum used in immunohistochemistry included mouse monoclonal antibody to CAPERα (Cat: ab56596, Abcam Inc., Cambridge, MA, USA), rabbit polyclonal antibody to VEGF (Cat: sc-152, Santa Cruz Biotechnology, INC., Dallas, TX, USA), mouse monoclonal antibody to CD34 (Cat: sc-74499, Santa Cruz Biotechnology, INC., Dallas, TX, USA).

Antigen retrieval was performed by microwaving the slides for 30 sec in citrate-based antigen retrieval solution (BioGenex, San Ramon, CA, USA). The sections were incubated overnight at 4°C with mouse monoclonal anti-CAPERα antibody (1:1000), rabbit polyclonal anti-VEGF antibody (1:300) or mouse monoclonal anti-CD34 (1:100) at the recommended dilutions and rinsed in PBS three times. Biotinylated secondary antibody, ABC (Avidin: Biotinylated enzyme complex), and DAB (3,3′-diaminobenzidine) substrate were used as detecting reagents according to the manufacturer's recommendation (Vector Laboratories, Burlingame, CA, USA). The slides were counterstained with hematoxylin, fixed in Scott's solution and dehydrolyzed with different concentrations of ethanol and Citrisolvent. Finally, the slides were mounted with permount mounting medium and observed under the microscope.

Antigen staining was evaluated semi-quantitatively based on Yu's method [16]. Whole high-power fields (400× magnification) for each tissue section were analyzed. For each antigen, Positive Rate (PR) and Staining Intensity (SI) were used to describe the expression of antigens.

### Statistical analysis

Statistical analysis was performed using the SPSS 19.0 software package. The chi-square (χ^2^) test was used to compare frequency of antibody against CAPERα among different groups, and degree of expression of CAPERα, VEGF and CD34 in normal liver tissues and HCC tissues. The Kruskal-Wallis test was used to compare the expression level (PR+SI) of CAPERα, VEGF and CD34 among normal liver, HCC, HCC grade II and HCC grade III tissues. Spearman's rank-order test was used to assess the correlations among the expression level (PR+SI) of CAPERα, VEGF and CD34. All statistical tests are two-sided.

## Abbreviations

AP-1: activating proein-1
ASC-1: activating signal cointegrator
CAPERα: coactivator of activating protein 1 and estrogen receptors
ELISA: Enzyme-linked immunosorbent assay
ER: Estrogen receptor
HCC: Hepatocellular carcinoma
IHC: Immunohistochemistry
TAA: tumor-associated antigen
TAD: transcription activation domain
VEGF: vascular endothelial growth factor

## References

[1] Jemal A, Bray F, Center MM, Ferlay J, Ward E, Forman D (2011). Global cancer statistics. CA Cancer J Clin.

[2] Fattovich G, Stroffolini T, Zagni I, Donato F (2004). Hepatocellular carcinoma in cirrhosis: incidence and risk factors. Gastroenterology.

[3] Imai H, Nakano Y, Kiyosawa K, Tan EM (1993). Increasing titers and changing specificities of antinuclear antibodies in patients with chronic liver disease who develop hepatocellular carcinoma. Cancer.

[4] Dai L, Ren P, Liu M, Imai H, Tan EM, Zhang JY (2014). Using immunomic approach to enhance tumor-associated autoantibody detection in diagnosis of hepatocellular carcinoma. Clin Immunol.

[5] Liu M, Zheng SJ, Chen Y, Li N, Ren PF, Dai LP, Duan ZP, Zhang JY (2014). Autoantibody response to murine double minute 2 protein in immunodiagnosis of hepatocellular carcinoma. J Immunol Res.

[6] Imai H, Chan EK, Kiyosawa K, Fu XD, Tan EM (1993). Novel nuclear autoantigen with splicing factor motifs identified with antibody from hepatocellular carcinoma. J Clin Invest.

[7] Jung DJ, Na SY, Na DS, Lee JW (2002). Molecular cloning and characterization of CAPER, a novel coactivator of activating protein-1 and estrogen receptors. J Biol Chem.

[8] Dowhan DH, Hong EP, Auboeuf D, Dennis AP, Wilson MM, Berget SM, O'Malley BW (2005). Steroid hormone receptor coactivation and alternative RNA splicing by U2AF65-related proteins CAPERalpha and CAPERbeta. Mol Cell.

[9] Dutta J, Fan G, Gelinas C (2008). CAPERalpha is a novel Rel-TAD-interacting factor that inhibits lymphocyte transformation by the potent Rel/NF-kappaB oncoprotein v-Rel. J Virol.

[10] Huang G, Zhou Z, Wang H, Kleinerman ES (2012). CAPER-alpha alternative splicing regulates the expression of vascular endothelial growth factor(1)(6)(5) in Ewing sarcoma cells. Cancer.

[11] Zhang JY, Megliorino R, Peng XX, Tan EM, Chen Y, Chan EK (2007). Antibody detection using tumor-associated antigen mini-array in immunodiagnosing human hepatocellular carcinoma. J Hepatol.

[12] Koziol JA, Zhang JY, Casiano CA, Peng XX, Shi FD, Feng AC, Chan EK, Tan EM (2003). Recursive partitioning as an approach to selection of immune markers for tumor diagnosis. Clin Cancer Res.

[13] Zhang J, Wang K, Liu SS, Dai L, Zhang JY (2011). Using proteomic approach to identify tumor-associated proteins as biomarkers in human esophageal squamous cell carcinoma. J Proteome Res.

[14] Looi K, Megliorino R, Shi FD, Peng XX, Chen Y, Zhang JY (2006). Humoral immune response to p16, a cyclin-dependent kinase inhibitor in human malignancies. Oncol Rep.

[15] Ersvaer E, Zhang JY, McCormack E, Olsnes A, Anensen N, Tan EM, Gjertsen BT, Bruserud O (2007). Cyclin B1 is commonly expressed in the cytoplasm of primary human acute myelogenous leukemia cells and serves as a leukemia-associated antigen associated with autoantibody response in a subset of patients. Eur J Haematol.

[16] Yu J, Ren X, Chen Y, Liu P, Wei X, Li H, Ying G, Chen K, Winkler H, Hao X (2013). Dysfunctional activation of neurotensin/IL-8 pathway in hepatocellular carcinoma is associated with increased inflammatory response in microenvironment, more epithelial mesenchymal transition in cancer and worse prognosis in patients. PLoS One.

[17] Tan EM, Zhang J (2008). Autoantibodies to tumor-associated antigens: reporters from the immune system. Immunol Rev.

[18] Winter SF, Minna JD, Johnson BE, Takahashi T, Gazdar AF, Carbone DP (1992). Development of antibodies against p53 in lung cancer patients appears to be dependent on the type of p53 mutation. Cancer Res.

[19] Labrecque S, Naor N, Thomson D, Matlashewski G (1993). Analysis of the anti-p53 antibody response in cancer patients. Cancer Res.

[20] Lubin R, Schlichtholz B, Bengoufa D, Zalcman G, Tredaniel J, Hirsch A, Caron de Fromentel C, Preudhomme C, Fenaux P, Fournier G, Mangin P, Laurent-Puig P, Pelletier G, Schlumberger M, Desgrandchamps F, Le Duc A (1993). Analysis of p53 antibodies in patients with various cancers define B-cell epitopes of human p53: distribution on primary structure and exposure on protein surface. Cancer Res.

[21] Yamaguchi R, Yano H, Iemura A, Ogasawara S, Haramaki M, Kojiro M (1998). Expression of vascular endothelial growth factor in human hepatocellular carcinoma. Hepatology.

[22] Choueiri TK, Figueroa DJ, Fay AP, Signoretti S, Liu Y, Gagnon R, Deen K, Carpenter C, Benson P, Ho TH, Pandite L, de Souza P, Powles T, Motzer RJ (2015). Correlation of PD-L1 tumor expression and treatment outcomes in patients with renal cell carcinoma receiving sunitinib or pazopanib: results from COMPARZ, a randomized controlled trial. Clin Cancer Res.

[23] Hlatky L, Hahnfeldt P, Folkman J (2002). Clinical application of antiangiogenic therapy: microvessel density, what it does and doesn't tell us. J Natl Cancer Inst.

[24] Pomme G, Augustin F, Fiegl M, Droeser RA, Sterlacci W, Tzankov A (2015). Detailed assessment of microvasculature markers in non-small cell lung cancer reveals potentially clinically relevant characteristics. Virchows Arch.

[25] Erbersdobler A, Isbarn H, Dix K, Steiner I, Schlomm T, Mirlacher M, Sauter G, Haese A (2010). Prognostic value of microvessel density in prostate cancer: a tissue microarray study. World J Urol.

[26] Murakami K, Kasajima A, Kawagishi N, Ohuchi N, Sasano H (2015). Microvessel density in hepatocellular carcinoma: Prognostic significance and review of the previous published work. Hepatol Res.

[27] Bialas M, Dyduch G, Dudala J, Bereza-Buziak M, Hubalewska-Dydejczyk A, Budzynski A, Okon K (2014). Study of microvessel density and the expression of vascular endothelial growth factors in adrenal gland pheochromocytomas. Int J Endocrinol.

[28] Kostis G, Ioannis L, Helen K, Helen P (2014). The expression of vascular endothelial growth factor-C correlates with lymphatic microvessel density and lymph node metastasis in prostate carcinoma: An immunohistochemical study. Urol Ann.

[29] Miyata Y, Mitsunari K, Asai A, Takehara K, Mochizuki Y, Sakai H (2015). Pathological significance and prognostic role of microvessel density, evaluated using CD31, CD34, and CD105 in prostate cancer patients after radical prostatectomy with neoadjuvant therapy. Prostate.

[30] Coston WM, Loera S, Lau SK, Ishizawa S, Jiang Z, Wu CL, Yen Y, Weiss LM, Chu PG (2008). Distinction of hepatocellular carcinoma from benign hepatic mimickers using Glypican-3 and CD34 immunohistochemistry. Am J Surg Pathol.

[31] Mesa RA, Hanson CA, Rajkumar SV, Schroeder G, Tefferi A (2000). Evaluation and clinical correlations of bone marrow angiogenesis in myelofibrosis with myeloid metaplasia. Blood.

[32] Gianelli U, Vener C, Raviele PR, Savi F, Somalvico F, Calori R, Iurlo A, Radaelli F, Fermo E, Bucciarelli P, Bori S, Coggi G, Deliliers GL (2007). VEGF expression correlates with microvessel density in Philadelphia chromosome-negative chronic myeloproliferative disorders. Am J Clin Pathol.

[33] Yang X, Sun HJ, Li ZR, Zhang H, Yang WJ, Ni B, Wu YZ (2015). Gastric cancer-associated enhancement of von Willebrand factor is regulated by vascular endothelial growth factor and related to disease severity. BMC Cancer.

[34] Tanigawa N, Lu C, Mitsui T, Miura S (1997). Quantitation of sinusoid-like vessels in hepatocellular carcinoma: its clinical and prognostic significance. Hepatology.

[35] Mise M, Arii S, Higashituji H, Furutani M, Niwano M, Harada T, Ishigami S, Toda Y, Nakayama H, Fukumoto M, Fujita J, Imamura M (1996). Clinical significance of vascular endothelial growth factor and basic fibroblast growth factor gene expression in liver tumor. Hepatology.

[36] Zhu AX, Duda DG, Sahani DV, Jain RK (2011). HCC and angiogenesis: possible targets and future directions. Nat Rev Clin Oncol.

[37] Bishayee A, Darvesh AS (2012). Angiogenesis in hepatocellular carcinoma: a potential target for chemoprevention and therapy. Curr Cancer Drug Targets.

[38] Carmeliet P, De Smet F, Loges S, Mazzone M (2009). Branching morphogenesis and antiangiogenesis candidates: tip cells lead the way. Nat Rev Clin Oncol.

[39] Llovet JM, Ricci S, Mazzaferro V, Hilgard P, Gane E, Blanc JF, de Oliveira AC, Santoro A, Raoul JL, Forner A, Schwartz M, Porta C, Zeuzem S, Bolondi L, Greten TF, Galle PR (2008). Sorafenib in advanced hepatocellular carcinoma. N Engl J Med.

[40] Tan EM (2012). Autoantibodies, autoimmune disease, and the birth of immune diagnostics. J Clin Invest.

